# The importance of estimating prevalence of ME/CFS in future epidemiological studies of long COVID

**DOI:** 10.3389/fpubh.2023.1275827

**Published:** 2023-11-03

**Authors:** Anna D. Grabowska, Francisco Westermeier, Luís Nacul, Eliana Lacerda, Nuno Sepúlveda

**Affiliations:** ^1^Department of Biophysics, Physiology, and Pathophysiology, Medical University of Warsaw, Warsaw, Poland; ^2^Department of Health Studies, Institute of Biomedical Science, FH Joanneum University of Applied Sciences, Graz, Austria; ^3^Centro Integrativo de Biología y Química Aplicada (CIBQA), Universidad Bernardo O'Higgins, Santiago, Chile; ^4^Women's Health Research Institute and BC Women's Hospital, University of British Columbia (BC), Vancouver, BC, Canada; ^5^Department of Clinical Research, Faculty of Infectious and Tropical Diseases, London School of Hygiene and Tropical Medicine, London, United Kingdom; ^6^Faculty of Mathematics & Information Science, Warsaw University of Technology, Warsaw, Poland; ^7^CEAUL – Centro de Estatística e Aplicações da Universidade de Lisboa, Lisbon, Portugal

**Keywords:** ME/CFS, long COVID, prevalence, diagnostic criteria, fatigue, post-exertional malaise, disease duration, unrefreshing sleep

## 1. Introduction

The end of the COVID-19 pandemic is generating a wide interest on long COVID (LC) (1), a heterogeneous medical condition known by many alternative names, such as post-COVID-19 syndrome (2), post-acute COVID-19 syndrome (3), and post-acute sequelae of SARS-CoV-2 infection (4), and persistent post-COVID-19 syndrome (5). This condition is a top priority in the current biomedical research agenda due to its great impact on public health (6). The clinical manifestation of LC varies from mild and temporary symptoms, such as anosmia and ageusia, to highly debilitating and chronic fatigue and post-exertional malaise (PEM) (7). This spectrum of symptoms might be explained by immune dysregulation, microbiota dysbiosis, autoimmunity and immune priming, abnormal blood clotting and endothelial-related problems, and neurological signaling dysfunction, among other pathological mechanisms (1, 8).

The real burden of LC remains elusive even though systematic reviews aggregate data from hundreds of studies and thousands of individuals (9–11). The underlying problems are the reliance on self-reporting of symptoms for the LC diagnosis and the challenge of conducting studies without any sources of sampling bias (10). The same problems emerge in the few epidemiological studies on Myalgic Encephalomyelitis/Chronic Fatigue Syndrome (ME/CFS) (12, 13). This disease remains without a specific biomarker (14), but might share some pathological mechanisms and symptoms with LC (4, 15–18). According to a recent meta-analysis (13), the pooled estimate of ME/CFS prevalence across multiple studies is 0.89% [95% CI = (0.60%−1.33%)]. This estimate shows some variations according to gender (1.36% in women vs. 0.86% in men), age (0.65% in adults vs. 0.55% in children and adolescents), or study setting (0.76% in community-based study vs. 0.63% in primary care studies). This disease inflicts dramatic individual and societal costs, such as health deterioration, reduced productivity, earnings and employment, mental health problems, and burnout (19).

Given the global urgency of managing and treating LC, several studies (20–23) are combining basic research on this disease and ME/CFS with the idea of accelerating knowledge of the underlying pathological mechanisms. However, similar combined approach remains to be adopted in epidemiological studies of LC. Therefore, these studies could expand their objectives to include the estimation of ME/CFS prevalence as well. These additional data offer a better quantification of the real burden of LC due to people that develop ME/CFS-related symptoms. Such a quantification provides the foundation for using consensual guidelines for ME/CFS healthcare to LC case management (15) that could be adopted and adapted for the specificities of a given national health system.

Such an expansion of objectives comes at a minimal cost by simply incorporating standard symptom questionaries used for the ME/CFS diagnosis and then running a diagnostic algorithm based on consensual case definitions. With this in mind, we reviewed the most consensual case definitions of ME/CFS. We also compared the symptoms assessed in the UK ME/CFS Biobank (UKMEB) (24, 25) with those documented in recent LC epidemiological studies. Finally, we provided some practical recommendations for future studies.

## 2. Brief review of ME/CFS and LC diagnostic criteria

ME/CFS has more than 20 proposed case definitions (26, 27). Among these definitions, the 1994 CDC (28), the 2003 Canadian Consensus Criteria (CCC) (29), and the 2015 Institute of Medicine (IOM) criteria have been used as diagnostic tools for research purposes (30). These criteria are also used for patients' enrollment in the UKMEB (24, 25).

The 1994 CDC criteria is mainly a research tool for ME/CFS diagnosis. In these criteria, an individual receives an ME/CFS diagnosis if she (or he) experiences unexplained, persistent, or relapsing fatigue for at least 6 months. The fatigue experienced should substantially reduce the normal levels of daily activities. Resting is also insufficient to restore normal energy levels. The individual should also experience four or more of the following eight symptoms:

I Substantial impairment in short-term memory or concentration;
II Sore throat;
III Tender cervical and axillary lymph nodes;
IV Muscle pain;
V Multi-joint pain without swelling or redness;
VI Headaches of a new type, pattern, or severity;
VII Unrefreshing sleep;
VIII PEM.

Note that a group of experts recommend PEM as a hallmark rather than an optional symptom to consider the diagnosis of ME/CFS based on these criteria (30). Exclusion criteria include all medical conditions that could explain fatigue (e.g., untreated hypothyroidism or sleep apnea), alcohol or other substance abuse, and severe obesity (body mass index greater than 45 kg/m^2^). Other authors discussed the possibility of defining a severely obese individual by a body mass index equal to or greater than 40 kg/m^2^ (31).

The 2003 CCC is basically a diagnostic tool for clinical settings. However, many research studies are using this tool for ME/CFS diagnosis. This criterion also recognizes 6 months as the minimal symptom duration. The hallmark symptoms are pathological fatigue, PEM, sleep abnormalities (unrefreshing sleep, reduced sleep quality or quantity, reversed or chaotic diurnal sleep rhythms), muscle or multi-joint pain, and two or more cognitive symptoms. The diagnosis also requires the presentation of one or more symptoms belonging to at least two additional domains: autonomic, neuroendocrine, and immune. Exclusion criteria also apply.

The 2015 IOM criterion is also a primary diagnostic tool for clinical settings. It also requires the presence of fatigue for more than 6 months, PEM, and unrefreshing sleep. This case definition also requires at least one of the following manifestations: cognitive impairment and orthostatic intolerance. In contrast to the 1994 CDC and 2003 CCC criteria, the 2015 IOM criterion does not contemplate any list of exclusionary medical conditions or co-morbidities. However, one should not diagnose a patient as having ME/CFS if treatment for the alternative diagnosis eliminates all symptoms in a patient. A recent discussion about the exclusionary medical conditions can be found elsewhere (32).

According to the World Health Organization, the definition of LC is the presence of at least one unresolved symptom after 3 months of a confirmed SARS-CoV-2 infection (2). Another definition is based on an international Delphi consensus of 11 outcomes for the core symptom set of LC (33). These outcomes are: fatigue; pain; post-exertion symptoms; work or occupational and study changes; survival; and functioning, symptoms, and conditions for each of cardiovascular, respiratory, nervous system, cognitive, mental health, and physical outcomes. Alternatively, the LC diagnosis might be diagnosed by a disease scoring system based on 37 symptoms (34).

## 3. Symptoms reported by patients in UKMEB and in current LC prevalence studies

Given the broad clinical spectrum of LC patients, prevalence studies of LC typically estimate the individual prevalence of a large number of symptoms. The basic question is whether these studies collect symptom data that would also allow them conducting a possible diagnosis of ME/CFS in the study participants.

Previously, we reported the prevalence of each of 47 symptoms evaluated in 222 ME/CFS patients upon their enrollment in the UKMEB, as reported elsewhere (35). Excluding fatigue (with 100% prevalence), the prevalence per symptom varied from 33.9% (palpitations) to 98.7% (unrefreshing sleep) with an average prevalence of 72.0%. Therefore, we can conclude that these 47 symptoms are highly prevalent in patients with ME/CFS complying with the 1994 CDC or the 2003 CCC criteria.

We then investigated whether three large systematic reviews of LC prevalence reported these symptoms. Besides the prevalence of fatigue (data not shown), there were only individual prevalence estimates for 23, 18, and 14 of the 47 UKMEB-related symptoms reported by O'Mahoney et al. (9), Woodrow et al. (10), and Natarajan et al. (11), respectively (Table 1). More importantly, these reviews did not report data related to PEM, although post-exertion symptoms are in the core outcome set of LC (33). Besides that, two large survey reported the prevalence of PEM higher than 80% in LC patients (7, 34). This lack of reporting indicates that current LC epidemiological studies have not collected sufficient symptom data to allow for a preliminary symptom assessment necessary for an ME/CFS diagnosis.

**Table 1:** Forty-seven symptoms in the UKMEB symptom assessment questionnaire (based on 2003 CCC) and their reporting in three systematic reviews on the prevalence of LC and its symptoms.

**Domain**	**Description**	**UKMEB prevalence (%)**	**Prevalence in percentage (95% CI); number of studies (** ***n*** **)**		
			**O'Mahoney et al. (9)**	**Woodrow et al. (10)**	**Natarajan et al. (11)**
Autonomic	Air hunger, difficulty breathing, or shortness of breath on exertion/activity	58.5	22.6 (18.3–27.4); *n* = 70 (dyspnea) 19.6 (8.8–38.0); *n* = 6 (exertional breathlessness)	14.9 (1.6–64.9); *n* = 78 (breathing problems)	21.5 (14.4–32.1); *n* = 17 (dyspnea)
	Bladder problems	56.7	2.1 (0.7–5.9); *n* = 5 (affected urinary system)	NR	NR
	Dizziness	68.3	6.2 (3.5–10.8); *n* = 15	7.4 (0.8–45.4); *n* = 26	9.1 (4.3–19.6); *n* = 7
	Paleness	49.6	NR	NR	NR
	IBS symptoms	78.1	6.4 (3.8–10.6); *n* = 13 (gastrointestinal symptoms) 3.4 (2.1–5.4); *n* = 21 (diarrhea) 2. (1.2–3.8); *n* = 16 (vomiting/nausea)	3.9 (0.4–28.8); *n* = 49 (nausea/vomiting)	7.8 (4.8–12.6); *n* = 5 (diarrhea) 1.2 (0.7–2.3); *n* = 3 (vomiting) 14.6 (1.7–124.5); *n* = 2 (diarrhea/vomiting)
	Intolerance to standing up	51.8	NR	NR	NR
	Feeling lightheaded	73.2	NR	NR	NR
	Palpitations	33.9	6.3 (4.5–8.7); *n* = 22	5.8 (1.2–24.5); *n* = 26	14.2 (7.1–28.2); *n* = 6
Immunological	Fever/Chills	57.6	2.2 (0.5–9.2); *n* = 13	1.9 (0.1–34.7); *n* = 24 (fever) 1.0 (0.0–98.8); *n* = 4 (chills)	3.1 (1.5–6.3); *n* = 9
	Flu symptoms	71.9	10.2 (7.4–13.8); *n* = 50 (cough) 4.54 (1.5–13.1); *n* = 7 (nasal symptoms)	7.4 (1.3–33.5); *n* = 52 (cough)	17.8 (13.3–23.9); *n* = 14 (cough)
	Frequent viral infections with long recovery periods	52.7	NR	NR	NR
	Worsen sensitivity to light	66.1	NR	NR	NR
	Sore throat	71.9	2.8 (1.8–4.3); *n* = 14	3.5 (0.6–17.1); *n* = 22	6.4 (3.0–13.6); *n* = 9
	Morning stiffness	71.0	NR	NR	NR
	Tender glands	75.4	NR	NR	NR
Neuroendocrine	Intolerance to extremes of heat/cold	74.6	NR	NR	NR
	Decreased sexual function or interest	57.1	NR	NR	NR
	Unusually sweaty	55.4	9.7 (5.7–16.0); *n* = 8 (sweating/night sweats)	NR	NR
	Worsening of symptoms post stress	89.3	NR	NR	NR
Neurocognitive	Back weakness	64.0	NR	NR	NR
	Brain fog or confusion	77.2	4.1 (1.6–10.1); *n* = 9	NR	NR
	Trouble concentrating	96.0	18.6 (13.4–25.2); *n* = 11 (poor concentration)	NR	20.2 (12.9–31.8); *n* = 5 (attention/concentration deficit)
	Difficulty retaining or recalling information	81.7	19.9 (15.8–24.7); *n* = 23 (impaired memory)	10.1 (0.8–60.2); *n* = 49 (cognitive or memory problems)	NR
	Difficulty understanding things/thinking clearly	82.6	17.1 (10.1–27.4); *n* = 13 (cognitive dysfunction)	10.1 (0.8–60.2); *n* = 49 (cognitive or memory problems)	28.8 (10.0–83.2); *n* = 3 (cognitive impairment)
	Disorientation	50.5	NR	NR	NR
	Eyesight disturbance (temporary)	60.7	6.3 (3.8–10.3); *n* = 8 (affected vision)	10.0 (0.0–96.5); *n* = 4 (eye problems)	NR
	Loss of balance or unsteadiness while standing, unable to focus the vision	73.7	3.8 (1.2–11.1); *n* = 5 (vertigo)	NR	NR
	Muscle discomfort	86.2	NR	NR	NR
	Muscle weakness	85.3	NR	10.2; 0.5–72.2; *n* = 21 (weakness)	NR
	Neck weakness	54.9	NR	NR	NR
	Poor coordination or unsteady movements (while walking)	62.1	14.8 (9.8–21.5); *n* = 14 (impaired walking/mobility)	NR	NR
	Sensitivity to light/noise	77.7	3.1 (1.7–5.6); *n* = 21 (affected hearing)	3.8; 0.2–45.0; *n* = 11	NR
	Short term memory problems	83.5	19.9 (15.8–24.7); *n* = 23 (impaired memory)	10.1 (0.8–60.2); *n* = 49 (cognitive or memory problems)	18.4 (11.7–28.9); *n* = 5 (memory deficit)
	Slow thinking	75.9	NR	NR	NR
	Tingling/numbness in arms and/or legs	69.6	6.2 (2.8–13.2); *n* = 6 (paresthesia)	11.3 (0.7–69.5); *n* = 14 (tingling or itching)	NR
Pain	Pain in chest or abdomen	77.2 (chest)	7.2 (5.2–9.8); *n* = 39 (chest) 4.0 (2.2–7.1); *n* = 10 (abdomen)	6.7 (0.9–35.8); *n* = 43 (chest) 3.7 (0.1–63.8); *n* = 15 (abdomen)	12.1 (6.1–24.0); *n* = 11 (chest) 9.2 (3.6–23.8); *n* = 3 (abdomen)
	Migraine/headaches	38.4 (migraines) 77.2 (headaches)	6.8 (4.9–9.4); *n* = 27	6.5 (0.6–45.6); *n* = 51 (headaches)	10.5 (5.3–20.5); *n* = 14 (headaches)
	Joint/muscle pain	55.4 (joint) 88.0 (muscle)	14.3 (8.0–24.1); *n* = 16 (joint) 10.3 (6.9–14.9); *n* = 28 (muscle)	10.6 (1.0–57.5); *n* = 61	28.2 (14.8–54.1); *n* = 5 (joint) 13.3 (7.5–23.7); *n* = 13 (muscle)
PEM	Intolerance to exercise	81.7	NR	NR	NR
	Fatigue/exhaustion after activity that would not cause fatigue before	96.4	NR	NR	NR
	Malaise after exertion, lasting >24 h	96.0	NR	NR	NR
	Marked physical/mental fatigue/exhaustion after minimal effort, lasting >24 h	77.7	NR	NR	NR
	Pain after exertion/effort, lasting >24 h	75.9	NR	NR	NR
	Worsening of symptoms after exertion/effort, lasting >24 h	91.1	NR	NR	NR
Sleep	Problems in sleep, quality of duration; insomnia	85.7	23.5 (18.1–29.8); *n* = 34 (affected sleep)	13.2 (1.2–64.9); *n* = 42 (sleep problems)	19.1 (12.4–29.4); *n* = 10 (sleep disturbance)
	Unrefreshing sleep	98.7	NR	NR	NR

O'Mahoney et al. (9) included 194 studies on SARS-CoV-2 infected individuals who confirmed and self-reported symptoms at least 28 days after infection onset (mean follow-up of 126 days). Woodrow et al. (10) included 120 studies in which SARS-CoV-2 infected individuals were followed up for 3–12 months. Natarajan et al. (11) reported a meta-analysis of 36 studies among LC patients.

NR, not reported.

These systematic reviews also did not provide any data for other highly prevalent ME/CFS-related symptoms in the patients from the UKMEB, such as unrefreshing sleep (98.7%), sensitivity to light or noise (77.7%), tender lymph nodes (75.5%), and intolerance to heat and cold (74.6%; Table 1).

## 4. Discussion

Since the beginning of the COVID-19 pandemic in early 2020, it became clear that many people who experienced a SARS-CoV-2 infection remained ill with a clinical manifestation consistent with ME/CFS. However, most of the epidemiological studies of LC ignored this fact and, therefore, they did not assess the presence and severity of cardinal symptoms of ME/CFS diagnosis. This is an unfortunate missed opportunity, especially, in what estimating ME/CFS prevalence is concerned. One can seize this opportunity by conducting a study prospectively. These studies should be clear in the LC case definition, criteria for an acute COVID-19 episode, and the duration of follow-up, because these aspects might influence the subsequent statistical results. Studies using a retrospective design as reported in recent meta-analyses of LC should be avoided, because they might miss crucial data (e.g., PEM) unavailable from routine healthcare records.

There is evidence for a limited assessment of symptoms in LC patients related to ME/CFS, including PEM, unrefreshing sleep, and sensitivity to light or noise in epidemiological studies of LC. Most of these unreported symptoms are auxiliary rather than strictly mandatory for an ME/CFS diagnosis, but they might be useful for defining disease subtypes (36, 37). The only exceptions are the PEM-related symptoms, which are key in the 2003 CCC, the 2015 IOM criteria, and in the modified 1994 CDC criterion (30). The assessment of PEM or other symptoms is becoming more important, given that the duration of LC can reach 3 years by now in some patients (6). In the case of ME/CFS, a 2-year disease duration might reflect the transition from an early to an established disease stage (38). Hence, it is conceivable that LC cases without typical symptoms of ME/CFS at an early disease stage might develop key symptoms of this disease when the chronicity of LC symptoms becomes established.

We recommend that future epidemiological studies of LC use the DePaul Symptoms Questionnaire (DSQ) (39) or the UKMEB Symptoms Assessment Questionnaire (35), as diagnostic tools enabling the identification of cases meeting commonly used diagnostic criteria for ME/CFS. Given the cardinal importance of PEM in ME/CFS diagnosis, one should make the effort to capture accurately its different aspects, such as recovery time, frequency, and severity (40). In this scenario, one can use the DSQ dedicated to PEM specifically, the so-called DSQ-PEM (41). The use of this questionnaire is likely to be more informative than simply asking for the presence of symptoms worse after even minor physical or mental effort, as done in large study of LC (34). The reporting of the epidemiological findings could be done via a recommended guideline for the minimal data elements on ME/CFS research (42). Besides typical information about study design and demographics, one should report the case definition used, the symptom inventory, the excluded medical and psychiatric conditions and co-morbidities, and self-reported functional impairment/levels of activity.

The major difficulty to comply with the 1994 CDC and the 2003 CCC in epidemiological studies lies in the exclusion of other medical conditions that could explain fatigue. The 2015 IOM criterion, alternatively, does not impose any exclusionary conditions (32), however, they still require a clinical assessment and consideration of differential diagnosis. This case definition is already being used to report the frequency of ME/CFS cases among LC cases (34, 43). However, the use of the 2015 IOM criteria might lead to inconsistent findings across studies due to variations in frequency of co-morbidities present in different populations. It might also overestimate the prevalence of ME/CFS due to highly-frequent conditions, such as diabetes and obesity in suspected cases. For example, 43% of participants fit the 2015 IOM criterion for ME/CFS in an LC study (43). Among these compliant individuals, some had a BMI of 45 kg/m^2^.

We also recommend raising the standard of research and reporting in LC, ME/CFS, and other chronic diseases; we made a similar recommendation for genetic association studies in ME/CFS (44). Our recommendation is based on two systematic reviews of LC prevalence data. One systematic review suggested that 45% of LC cases had ME/CFS (45). However, this review incorrectly assumed that the persistence of fatigue was equivalent to ME/CFS. The other systematic review suggested that only a few epidemiological studies collected representative samples from the LC population (10). Low population representativeness, convenience sampling, and different sources of bias might be present in the remaining published studies (10). Randomness and sample representativeness are the pillars of a sound statistical inference. If a study does not minimally ensure these foundational assumptions, the subsequent statistical inference might be tricky, or even impossible (46).

## Author contributions

ADG: Investigation, Validation, Writing – review & editing. FW: Investigation, Validation, Writing – review & editing. LN: Data curation, Resources, Writing – review & editing. EL: Data curation, Resources, Writing – review & editing. NS: Conceptualization, Investigation, Validation, Writing – original draft, Writing – review & editing.
